# Skull Base Reconstruction after Endonasal Endoscopic Resection of Synchronous Sinonasal Adenoid Cystic Carcinoma and Intracranial Meningioma

**DOI:** 10.1055/a-2837-3442

**Published:** 2026-04-07

**Authors:** Martin Jurlina, Boris Kos, Matija Mamić, Ivica Lukšić, Edward C. Kuan, Marica Žižić, Krsto Dawidowsky

**Affiliations:** 1 School of Medicine, University of Zagreb, Zagreb, Croatia; 2 Department of Maxillofacial Surgery, University Hospital Dubrava, Zagreb, Croatia; 3 Department of Otolaryngology-Head and Neck Surgery, University of California, Irving, Orange, California, United States; 4 Department of Otorhinolaryngology-Head and Neck Surgery, University Hospital Sveti Duh, Zagreb, Croatia; 5 Department of Otorhinolaryngology and Head and Neck Surgery, University Hospital Zagreb, Zagreb, Croatia

**Keywords:** malignant cranial base tumors, endoscopic dural reconstruction, free temporalis muscle fascia graft, endoscopic intracranial tumor resection

## Abstract

Synchronous skull base tumors have rarely been reported in the literature. The endoscopic approach has now become standard for most anterior skull base tumors. An open approach is considered in cases when an endoscopic approach is contraindicated or inadequate for full resection. Since tumor resection often results in significant dural defects, dural reconstruction represents critical part of endonasal endoscopic surgery. To prevent serious complications and achieve optimal outcomes, recreation of airtight and watertight barrier between the anterior cranial fossa and the sinonasal cavity and ensuring mechanical support are essential. The free fascia lata graft currently represents the gold standard for endonasal endoscopic dural reconstruction following tumor resection. Other options include free grafts (e.g., free mucosal grafts, free abdominal fat grafts, free temporalis muscle fascia grafts [FTMFG]), vascularized axial flaps (e.g., pericranial flaps, nasoseptal flaps, axial middle turbinate flaps), and synthetic materials. The use of FTMFG for dural reconstruction has anecdotally been reported only in open surgical approaches. In this technically challenging case, FTMFG was used for dural reconstruction following endoscopic resection in a rare case of synchronous extracranial malignant and extensive intracranial benign cranial base tumor. The preexisting communication justified simultaneous resection over skull base preservation. Negative margins were achieved 360 degrees around the defect ( Video 1 ).

**Video 1** Endoscopic endonasal skull base reconstruction following resection of synchronous sinonasal adenoid cystic carcinoma and intracranial meningioma.


